# Intramyocellular lipid kinetics and insulin resistance

**DOI:** 10.1186/1476-511X-6-18

**Published:** 2007-07-24

**Authors:** ZengKui Guo

**Affiliations:** 1 Endocrine Research Unit, Mayo Foundation, Rochester, Minnesota 55905, USA

## Abstract

More than fifteen years ago it was discovered that intramyocellular triglyceride (imcTG) content in skeletal muscle is abnormally high in conditions of lipid oversupply (e.g. high fat feeding) and, later, obesity, type 2 diabetes (T2D) and other metabolic conditions. This imcTG excess is robustly associated with muscle insulin resistance (MIR). However, to date the pathways responsible for the imcTG excess and the mechanisms underlying the imcTG-MIR correlation remain unclear. A current hypothesis is based on a backward mechanism that impaired fatty acid oxidation by skeletal muscle causes imcTG to accumulate. As such, imcTG excess is considered a marker but not a player in MIR. However, recent results from kinetic studies indicated that imcTG pool in high fat-induced obesity (HFO) model is kinetically dynamic. On one hand, imcTG synthesis is accelerated and contributes to imcTG accumulation. On the other, the turnover of imcTG is also accelerated. A hyperdynamic imcTG pool can impose dual adverse effects on glucose metabolism in skeletal muscle. It increases the release and thus the availability of fatty acids in myocytes that may promote fatty acid oxidation and suppress glucose utilization. Meanwhile, it releases abundant fatty acid products (e.g. diacylglycerol, ceramides) that impair insulin actions via signal transduction, thereby causing MIR. Thus, intramyocellular fatty acids and their products released from imcTG appear to function as a link to MIR. Accordingly, a forward mechanism is proposed that explains the imcTG-MIR correlation.

## Background

Intramyocellular triglyceride (imcTG) is an important energy source for skeletal muscle. In 1991, Storlien and associates reported that under high fat feeding, imcTG content is elevated to abnormally high levels and this is correlated with muscle insulin resistance (r = 0.86–0.95) [1]. The observation has been since confirmed in a number of other models of insulin resistance such as obesity, T2D and hypertension [2-7]. As skeletal muscle accounts for the bulk of insulin-mediated glucose disposal and thus is important for systemic energy metabolism, the implication of this imcTG-MIR correlation to health and disease is significant. This is exemplified by the ensuing active investigations on this subject [3,8-11]. The establishment and utilization of proton MRS technology for in vivo measurement of imcTG content was largely in response to these investigations [12-15]. However, most of the investigations have been largely correlative in nature.

Now it is increasingly realized that imcTG is the most robust correlate of MIR, stronger than other metabolic indicators such as % body fat, body mass index (BMI) and waist-hip ratio [2]. This suggested a more substantive role for imcTG in MIR, rather than merely a marker of MIR. However, investigations on the mechanism(s) underlying the imcTG-MIR correlation have been less fruitful than the studies of imcTG pool size so that it remains a phenomenon not well understood as to whether and how the enlarged imcTG pool is related to MIR. Glucose-fatty acid cycling (i.e. Randle cycle) was considered by some to explain, at least in part, the correlation due to the interactions between glucose and fatty acid metabolism (substrate competition) [16]. However, later the emphasis of the investigations seems to have shifted to the area of signal transduction. It has been shown that under conditions of lipid oversupply, obesity and other forms of insulin resistance, the content of signaling molecules, including long chain acyl CoA (LCACoA), diacylglycerol (DAG) and ceramides are increased in skeletal muscle [9]. These molecules interfere with insulin signaling via PKC system, thereby contributing to MIR [17-19]. Metabolic inflexibility theory has also been proposed to explain MIR in T2D [20].

By comparison, there have been limited research efforts on the kinetics of imcTG metabolism, and its role in MIR, such as synthesis, turnover and oxidation, the pathways that determine imcTG pool size. In the face of enlarged imcTG pool size, abnormality in imcTG kinetics may function as an independent factor to exert metabolic consequences by altering intramyocellular fatty acid metabolism, thereby contributing to MIR. This review is focused on the synthesis, turnover and utilization of fatty acids and their derivatives derived from the intramyocellular neutral lipid pool (imcTG) and their roles in MIR as related to obesity and T2D.

## imcTG synthesis

For imcTG content to increase, its synthesis must increase or its utilization decreases, or both. At present time, it is not clear whether it is the synthetic or utilization limb(s) that is responsible for the observed excess imcTG accumulation. Clarifying this question is important, however. If it is the utilization, then it would imply that imcTG excess is merely a marker (consequence) of impaired fatty acid utilization (β-oxidation). This is termed backward mechanism, meaning that reduced fatty acid oxidation acts backward to cause imcTG accumulation, the substrate source pool for β-oxidation. In contrast, if it is the synthesis, it would mean that imcTG is more actively involved as a source of MIR by accumulating excess amount of fatty acids in it. To maintain imcTG homeostasis, the enlarged imcTG pool turns over rapidly and thus releases abundant fatty acid products that interfere with insulin signaling. This is termed forward mechanism, meaning that imcTG is the source pool that causes the adverse consequences. For the purpose of pharmaceutical intervention of MIR, the two mechanisms mean different targets. The backward mechanism requires manipulation (to stimulate) of fatty acid utilization in order to normalize imcTG pool size and to eliminate fatty acid products that impair insulin signaling. In contrast, the forward mechanism requires modulation (to suppress) of imcTG turnover or hydrolysis to contain intracellular fatty acid flux and availability. Therefore, it is important to determine which mechanism is (more) responsible for MIR.

Toward this direction, we have observed that the rate of incorporation of glycerol and fatty acid precursors into imcTG (synthesis) is grossly accelerated in three muscle types, gastrocnemius, soleus and tibialis anterior, in HFO rats [21]. The rate of imcTG synthesis was 2 (soleus) to 5 (gastrocnemius) fold higher than in lean littermates after 3 months on a high fat diet (55% energy from lard) at age of 4 months. At age of 8 months after 7 months on the diet, the rate of imcTG synthesis further accelerated to 3 (soleus and gastrocnemius) to 5 (tibialis anterior) fold higher in HFO rats. However, the accelerated imcTG synthesis slowed down by age of 12 months and 11 months on the same diet (~2 fold higher, soleus and gastrocnemius). In absolute term, the rate of imcTG synthesis is the highest at age of 8 months (5 nmol glycerol incorporated/g wet wt·min) and lowest at 12 months of age (<1 nmol/g ww·min) while it is intermediate at 4 months (2–3 nmol/g·min). In all age groups, imcTG pool size was correlated with synthesis rate [21]. As supporting evidence, it has been reported that in the heart of diabetic rats, TG synthesis is also accelerated which contributes to imcTG excess [22]. These are direct evidence that biosynthesis plays a pivotal role in imcTG accumulation at least in this obesity model. However, pool size was not always a function of synthesis, suggesting roles of other pathways, such as imcTG turnover and fatty acid utilization (below). Therefore, imcTG excess is a result of imbalance between exaggerated influx (synthesis) and efflux (utilization) of fatty acids into and out of the pool, respectively. On the other hand, synthesis appears to play the leading role even if fatty acid oxidation is increased, as reported. In other words, increase in synthesis overruns increase in oxidation, thereby resulting in net imcTG accrual. However, this is not the case during aging. imcTG synthesis reaches the plateau at around 8 months and then declines substantially at mid life (assuming a life span of 24 months). This regression in synthetic activity coincides with decreased lipid utilization and mitochondrial functions [23] and is inversely related to aging-related increase in imcTG stores [24]. Therefore, it seems that imcTG excess is mainly a result of accelerated synthesis when young but of decreased lipid utilization during aging.

The accelerated imcTG synthesis in HFO is consistent with our observation that in these rats, >80%, compared to 50% in lean rats, of plasma palmitate traverses imcTG before being oxidized in gastrocnemius. This indirect oxidation of plasma fatty acids (by traversing imcTG pool first) may make imcTG synthesis falsely high when it is determined based on substrate incorporation as we did. This probably explains why imcTG pool size is not a precise function of its synthesis rate. This illustrates again the point that imcTG pool size is determined by the delicate balance between influx and efflux, but not by either alone. Nonetheless, a conclusion that can be drawn from these observations is that the kinetics of imcTG synthesis in the HFO model is greatly accelerated and it likely plays an important role in imcTG accumulation. So far, while likely involved, the quantitative role of circulating VLDL-TG fatty acids in imcTG synthesis has not been studied.

It is important to point out that the accelerated synthesis in the HFO model was not a result of direct (high fat) dietary effects as plasma fatty acids, glycerol, triglycerides and glucose were all experimentally matched between lean and obese groups by substrate co-infusion [21]. Therefore, acceleration in imcTG synthesis is a phenotypic characteristic of obesity. This is in contrast with the assumption that imcTG excess is a result of reduced fatty acid utilization [25,26]. It is important to note that the assumption is largely based on the activities of enzymes involved in lipid oxidation such as CS (citrate synthase) and CPT1 (carnitine palmitoylacyltransferase-1), which are often discrepant from actual substrate flux. In fact, a large body of literature showed that lipid oxidation by skeletal muscle in obesity is actually increased [27,28].

## imcTG turnover

The study of imcTG turnover kinetics is very limited and no such studies seem to have been reported in relation to MIR. The limited reports indicated that in resting healthy humans, imcTG pool turns over slowly with a fractional rate of 0.0026/min (calculated from the reported rate, 29 h/pool) [29]. By using pulse (to prelabel imcTG pool with ^14^C-palmitate)-chase (to monitor the decay rate of the incorporated tracer) technique, we have determined that in young and healthy men and women exercising at 45% of VO_2max_, the fractional turnover rate (FTR) of imcTG was 0.0032 ± 0.0007/min [30]. By comparison, as expected, in rodents imcTG turns over much faster. In resting lean rats, FTR of imcTG was determined to be 0.013 ± 0.005/min, 0.016 ± 0.005/min and 0.0072 ± 0.003%/min for gastrocnemius, tibialis anterior and soleus muscle, respectively. In HFO rats, FTR of imcTG is even higher (0.026 ± 0.002/min for gastrocnemius, P = 0.02 and 0.030 ± 0.002/min for tibialis anterior, P = 0.01) compared to the values in the lean [31]. However, the obesity-related acceleration in imcTG turnover was not observed for soleus muscle, perhaps reflecting its larger pool size and thus lower FTR.

These findings are in contrast to the view that in obesity and T2D imcTG is a static pool turning over slowly that contributes to its excess accumulation [25]. The data reported for HFO rats indicated that it is clear that imcTG pool is not only enlarged but also kinetically hyperdynamic, meaning that its synthesis and turnover are both accelerated as compared to lean control. As turnover is a reflection of 2 opposing pathways, synthesis and lipolysis, the data suggested that these pathways are both accelerated several folds in HFO. This implies that imcTG recycles rapidly (imcTG → fatty acids → acyl CoA → imcTG). In humans, imcTG recycles constantly at least during exercises [30].

The accelerated imcTG dynamics, compounded by its enlarged pool size, translates into remarkable increases in the release of fatty acids from it. This results in increased fatty acid trafficking and availability in myocytes. Indeed, we routinely observed increases in intramyocellular non-esterified fatty acids (NEFA) in the HFO rats compared to lean control (gastrocnemius by 70%, soleus by 89% and extensor digitorus longus by 106%, all P < 0.01). Under the action of acyl CoA synthase, long chain fatty acids are rapidly activated to long chain acyl CoA. They are precursors to several pathways including mitochondrial β-oxidation and signal transduction.

## imcTG-fatty acid oxidation

An immediate fate for long chain acyl CoA is β-oxidation in mitochondria after being converted to long chain acylcarnitines catalyzed by CPT1 (the required form of long, but not medium or short, chain fatty acids to enter mitochondria). Peroxisomal β-oxidation is minor as the pathway primarily functions to shorten very long fatty acids for further oxidation by mitochondria [32,33]. Theoretically, by mass action alone, increased fatty acids may accelerate the cascade and thus mitochondrial β-oxidation. The close vicinity between imcTG droplets and mitochondria in myocytes [34] further facilitates this process. In fact, this is supported by experimental evidence. It has been extensively reported that lipid oxidation in obesity and T2D is increased [35,36]. Our preliminary results from in vivo studies in HFO rats confirmed this (unpublished data). Fatty acid oxidation is known to interfere with glucose metabolism through substrate competition [37], a well established mechanism under certain metabolic conditions substantiated by a large body of literature [27,28,38,39]. Therefore, a hyperdynamic imcTG pool can interfere with glucose metabolism at substrate level independently. The role of signal transduction, involving diacylglycerol and ceramides and perhaps long chain acyl CoA, via the PKC/IRS-1/PIK3 axis, in MIR can presumably supplement, but not substitute, this possibility. For example, substrate competition and signaling transduction may operate simultaneously. There are no reports whether they antagonize or are exclusive to each other. Experiments designed to test this hypothesis would be interesting and important for understanding of the interactions between signaling and β-oxidation pathways.

## imcTG-fatty acid signaling

NEFA and their derivatives (acyl CoA, diacylglycerol and ceramides) are also signaling molecules. Therefore, increased intramyocellular fatty acid availability may exert additional effects on muscle metabolism via signal transduction. Now it is known that the content of these molecules in skeletal muscle is increased with lipid oversupply and in obesity [17]. They activate PKCα,θ,ε isoforms which in turn phosphorylates serine/threonine residues of insulin receptor (IR) and insulin receptor substrate-1 (IRS-1) [9,17]. This in turn inhibits the activation of IR and IRS-1 (tyrosine phosphorylation), thereby impairing insulin signaling with retarded GLUT4 translocation to the plasma membrane compartment and hence reduced insulin-mediated glucose uptake (MIR).

The turnover of imcTG releases 1,2-diacylglycerol [40] and other isomers (1,3- and 2,3-). One diacylglycerol and one acyl CoA molecule (catalyzed by acyl CoA synthase) are produced from the hydrolysis of each imcTG molecule. The combined effects of these molecules may be powerful in inhibiting insulin signaling. We have observed that in HFO rats, the content of diacylglycerol in gastrocnemius, soleus and extensor digitorus longus is 52% (P < 0.01), 37% (P < 0.05) and 88% (P < 0.01) higher than in lean control, and is positively correlated with imcTG pool size and imcTG turnover rate. These observations suggest a precursor-product relationship between imcTG and diacylglycerol. Furthermore, imcTG as a source of these molecules has been confirmed using tracer techniques. Using ^14^C-glycerol, increased production of diacylglycerol from imcTG in gastrocnemius and soleus muscles of HFO rats has been observed compared to lean control. Therefore, an enlarged pool size and accelerated turnover of imcTG perhaps act in concert to increase the production of signaling molecules.

While diacylglycerol is a classical second messenger, whether the thioester form (acyl CoA) or the free form of fatty acids is the active form for signaling seems unclear. Contrasting to many other reports, Forman et al reported that the carboxyl group, -COOH, is required for the signaling activity and upon thioesterification, the activity is lost [41]. Nonetheless, as the two forms can interchange readily through enzymatic actions, this may not be a critical issue regarding the role of fatty acid signaling in MIR.

Although elevated long chain acyl CoA and diacylglycerol in conditions of lipid oversupply and insulin resistance have been extensively reported [17,19,42,43], the roles of imcTG turnover in this process have not been directly studied. Clarifying this question is important. If rapid imcTG turnover increases the production and the content of these signaling molecules, then it can be concluded that imcTG turnover kinetics contributes to MIR. In other words, rapid turnover functions as a link between imcTG and MIR. If so, imcTG is a source, not only a marker, of MIR. Therefore, turnover pathway of imcTG (e.g. hydrolysis) can be used as a target for the intervention of MIR by manipulating intracellular fatty acid dynamics and availability. Studies designed to quantify the flux of signaling molecules from imcTG pool may provide the needed information for pursuing in this direction.

Fatty acids are also precursor to ceramides, yet another member of the same signaling molecule family with similar functions. Thus, increased fatty acid availability can promote ceramide synthesis, further impairing insulin signaling.

**Figure 1:**
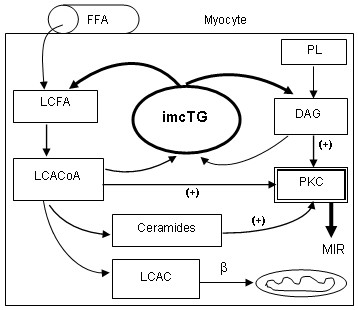
Hydrolysis of imcTG produces DAG and LCACoA which is also precursor to ceramides. All three can activate PKC isoforms that inhibit insulin signaling. An enlarged and rapidly turning over imcTG pool increases the release of these fatty acid derivatives, thus causing MIR. Simultaneously, mitochondrial β-oxidation may increase given the large fatty acid flux thereby worsening MIR. FFA, (plasma) free fatty acids; LCFA, long chain fatty acids; LCACoA, long chain acyl CoA; LCAC, long chain acylcarnitines; PL, phospholipids; DAG, diacylglycerol; PKC, protein kinase C; β, mitochondrial β-oxidation.

Collectively, long chain acyl CoA, diacylglycerol and ceramides can all be produced from imcTG. When imcTG pool becomes enlarged, the fluxes are increased. Hyperdynamic turnover amplifies and worsens this mass effect. As a result, excess amounts of signaling molecules are produced that activate PKC isoforms and inactivate PI3 kinase, thereby resulting in MIR (Fig 1).

Given the clear evidence of inhibitory effects of these molecules on insulin signaling, conceptually reduced fatty acid oxidation can further worsens glucose metabolism when more fatty acids become available for signaling. However, this may not necessarily be the case in that increased fatty acid oxidation may not be able to effectively divert fatty acids away from signaling pathways because of the great availability of fatty acids released from imcTG that may suffice for both pathways. In such case, glucose metabolism is subject to dual suppressions. On one hand, insulin-mediated glucose uptake is attenuated as a result of impaired insulin signaling (e.g. postprandial). On the other hand, glucose uptake is reduced via substrate competition due to increased fatty acid oxidation (e.g. postabsorptive). This may be the case when muscle lipid oxidation in obesity and type 2 diabetes is elevated [27,28,44,45]. However, to accept this scenario requires direct demonstration that both fatty acid oxidation and the signaling pathways are active. Nonetheless, it is clear that obesity is associated with a cluster of imcTG abnormalities including enlarged pool size, accelerated turnover, increased oxidation and production of fatty acids and their derivatives as signaling molecules. The metabolic effects of this cluster appear to link imcTG to MIR. This supports a forward mechanism where imcTG is a source of MIR. However, the mechanism may operate in overweight or non-severe obesity where lipid oxidation by muscle is likely high, but perhaps not in severe obesity where muscle lipid oxidation is low [8]. In severe obesity and during aging, the backward mechanism likely dominates.

## imcTG kinetics and MIR in humans

Studies of imcTG kinetics in humans are rare and virtually lacking as related to obesity, T2D or other metabolic diseases. Limited data are only available for healthy humans where imcTG turns over at 0.26%/min at rest [29] and 0.32%/min during modest exercises [30]. Therefore, it is not clear currently whether the forward mechanism is relevant in human obesity. Clearly, investigations on imcTG kinetics as related to MIR are required. On the other hand, based on the extensive reports on increased lipid oxidation by skeletal muscle in obesity and perhaps to a lesser extent in T2D, it is reasonable to estimate that the turnover of intramyocellular fatty acids, mainly of imcTG origin, is perhaps also accelerated as it is a prerequisite for increased oxidation (permissive role). For example, increases in oxidation are impossible without increase in fatty acid release from imcTG. Indeed, turnover and oxidation are often quantitatively related [46,47].

Overall, animal studies on imcTG kinetics are limited as well. By comparison, studies on heart triglyceride kinetics have been more active. Similar to that observed for HFO rats, heart triglyceride synthesis and turnover and fatty acid oxidation are all accelerated in diabetic rats [22,48,49]. Therefore, it appears that both obesity and diabetes are associated with hyperdynamics of intracellular triglyceride metabolism which likely contributes to MIR and cardiomyopathy. This supports the forward mechanism.

## Aging and MIR

As discussed above, in middle-aged HFO rats imcTG synthesis remains accelerated but attenuation was apparent compared to younger age. This pattern of changes in imcTG synthesis is consistent with the known aging-related functional regressions of mitochondria in humans, such as declines in biogenesis, oxidative capacity and oxidation-phosphorylation [50,51]. As a result, mitochondrial β-oxidation is reduced [23,52]. To maintain metabolic homeostasis, this is counter-balanced by reduction in imcTG synthesis. However, imcTG may continue to accumulate in excess [51] as a result of imbalance (i.e. reduction in utilization overruns reduction in synthesis). In this sense, imcTG excess is a random event as indicated by the fact that imcTG is not always increased in all insulin resistant states. This scenario confers with the backward mechanism where impaired lipid utilization causes imcTG to accumulate [23]. The mechanism seems also relevant to T2D which is associated with mitochondrial dysfunction along with MIR and imcTG excess [50]. This hypothesis seems more plausible considering that T2D develops more likely with aging and at the late stage of insulin resistance associated with obesity. Thus, in T2D as well as in aging, imcTG excess is a result, rather than source, of MIR.

**Figure 2:**
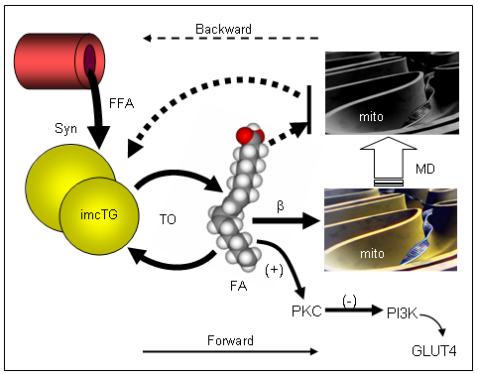
Schematic representation of forward (solid arrows) and backward (broken arrows) mechanism underlying MIR, as discussed. By the forward mechanism, imcTG is at a hyperdynamic state characterized by accelerated synthesis (Syn, ref. 21) and turnover (TO, ref. 31) in the earlier stages of metabolic complications such as obesity. This increases the flux and availability of intramyocellular fatty acids (FA/LCFA), DAG, LCACoA and ceramides and thus signaling via PKC system, and promotes mitochondrial β-oxidation (β). As a result, PI3K is inhibited and GLUT4 translocation impaired resulting in reduced insulin-mediated glucose uptake (MIR). Over time, the overloading of mitochondria by β-oxidation gradually causes mitochondrial damages and dysfunctions (MD) via mechanisms such as oxidative stress and DNA damage. When this occurs, mitochondrial β-oxidation reduces. At this stage (e.g. T2D or advanced aging), the backward mechanism prevails. The decline in fatty acid oxidation causes imcTG to accumulate. Gray-scaled mitochondria (mito) represents damaged mitochondria. The thickness of arrows approximates the sizes of the fluxes.

However, the backward mechanism does not appear to explain imcTG excess for other types of insulin resistance such as simple (non-diabetic) and non-severe obesity. Rather, the forward mechanism is more relevant to obesity at relatively young ages where insulin resistance is relatively mild without apparent alterations in mitochondrial structure, morphology or functions as seen in severe obesity, T2D or advanced aging. Indeed, pathological changes in subsarcolemmal mitochondria is more advanced in T2D than in obesity [53]. Instead, imcTG hyperdynamics is the predominant abnormality in obesity at early stage or younger ages associated mainly with signaling via PKC/PI3K systems and/or increased mitochondrial β-oxidation. These conditions perturb (overload) mitochondrial functions via mechanisms such as oxidative stress and inflammation. From these abnormalities, mitochondrial dysfunctions and structural alterations can gradually develop (e.g. mtDNA damages, ref. [54]). As this process continues, accompanied by aging, the forward mechanism reverts to backward where mitochondrial dysfunctions limit β-oxidation causing imcTG to accumulate. Therefore, the forward mechanism underlies insulin resistance at earlier stages of obesity whereas the backward mechanism prevails at later stages of insulin resistance such as T2D and advanced aging (Fig 2).

## New developments

The research on MIR evolves rapidly and some intriguing observations have been reported recently. Increasing imcTG pool size purposely by stimulating synthesis via genetic manipulation surprisingly improved insulin sensitivity and prevented high fat-induced insulin resistance [55]. This echoes a previous report that promoting fatty acid incorporation into imcTG reduced lipid toxicity in myocytes [56]. These findings appear to suggest that eliminating/reducing fatty acid products in myocytes prevents lipotoxicity and improves insulin sensitivity. This is complementary to the observation that promoting fatty acid oxidation reduced fatty acid products in skeletal muscle and improved insulin sensitivity [18]. Promoting imcTG synthesis also improved insulin sensitivity by protecting against inflammation [57]. Collectively, these studies essentially demonstrated that clearing up fatty acid products in myocytes using different approaches (e.g. pooling into imcTG or eliminating via oxidation) has the same effect of protecting cells against adverse effects. This is consistent with the fact that provision of fatty acids through plasma, which drastically increases intramyocellular fatty acid levels, rapidly induces MIR (within hours). Therefore, it appears that fatty acids and their derivatives that exist in intracellular compartments other than imcTG are a source of lipotoxicity and MIR and thus 'eliminating' them improves or protects insulin sensitivity.

## Summary

Recent findings suggested that intramyocellular triglyceride excess is a source, not only a marker, of muscle insulin resistance. The hyperdynamic turnover kinetics and enlarged pool size of imcTG function to release abundant fatty acids and their products known to interfere with insulin signaling and glucose metabolism via signal transduction and/or substrate competition. As such, imcTG hyperdynamics appears to be a link to MIR, as described by a forward mechanism. Alternatively, muscle insulin resistance and mitochondrial dysfunction in advanced obesity, T2D and aging may reverse the course causing imcTG to accumulate, as represented by a backward mechanism. Clarifying the mechanisms has implications to the understanding and potential intervention of muscle insulin resistance. For example, with the forward mechanism, the intramyocellular triglyceride pool is the target whereas it is only a surrogate with the backward mechanism.

## Abbreviations

BMI, body mass index

DAG, diacylglycerol

FFA, free fatty acids

HFO, high fat induced obesity

imcTG, intramyocellular triglycerides

LCAC, long chain acylcarnitine

LCACoA, long chain acyl CoA

LCFA, long chain fatty acids

MIR, muscle insulin resistance

MRS, magnetic resonance spectroscopy

NEFA, non-esterified (free) fatty acids

PL, phospholipids

PKC, protein kinase C

RBP4, retinol binding protein-4

T2D, type 2 diabetes

VLDL, very low density lipoprotein
